# Validity of the short physical performance battery for screening for frailty syndrome among older people in the Brazilian Amazon region. A cross-sectional study

**DOI:** 10.1590/1516-3180.2020.0264.R1.14092020

**Published:** 2020-11-27

**Authors:** Laila Lira Guimarães Rocco, Tiótrefis Gomes Fernandes

**Affiliations:** I MSc. Physiotherapist, Postgraduate Program on Health, Society and Endemics in the Amazon, Universidade Federal do Amazonas (UFAM), Manaus (AM), Brazil.; II PhD. Professor, School of Physical Education and Physiotherapy, Universidade Federal do Amazonas (UFAM), Manaus (AM), Brazil.

**Keywords:** Aging, Physical functional performance, Frailty, Reproducibility of results, Biological aging, Physical performance, Frailty syndrome, Validity, Amazon

## Abstract

**BACKGROUND:**

Environmental and population characteristics seem to influence the variation in cutoff points of the Short Physical Performance Battery (SPPB) for diagnosing frailty syndrome among older adults.

**OBJECTIVE:**

To verify the validity of the SPPB for screening for frailty syndrome among older adults in the Amazonian context.

**DESIGN AND SETTING:**

Cross-sectional population-based study on older adults in the urban area of Coari (AM), Brazil.

**METHODS:**

In total, 264 older adults (60 years of age or over) were included. Frailty syndrome was defined using the Fried phenotype criteria. The SPPB cutoff points were compared in relation to frailty and validity measurements were calculated for the test.

**RESULTS:**

A strong association between poor physical performance and frailty was identified (P < 0.001). The cutoff point of 6 demonstrated the best validity measurements for frailty in the sample studied (sensitivity: 0.28; specificity: 0.94; accuracy: 0.88; area under the receiver operating characteristic curve, AUC-ROC: 0.61; likelihood ratio, LR+: 4.44; LR-: 0.77; prevalence: 8.3%; post-test probability, PTP+: 0.32; PTP-: 0.07), with emphasis on high specificity and the positive likelihood ratio value.

**CONCLUSION:**

The SPPB was shown to be useful for screening frail older adults in the Amazon region. The score of 6 demonstrated the best cutoff point for this population. This could be used in healthcare services for diagnostic screening for frailty among older people within the Amazonian context.

## INTRODUCTION

Among the conditions attributed to aging, frailty syndrome (FS) is among the main ones and is associated with functional decline, hospitalization and early death. FS is defined as a clinical syndrome of spiraling energy decline, of multifactorial nature, based on a trio of alterations: sarcopenia, neuroendocrine dysregulation and immune dysfunction. It has repercussions on individuals' ability to achieve homeostatic adaptation, thus leading to a state of increased physiological vulnerability in the presence of stressors.^1^

Identification of FS among older adults is essential, for appropriate prevention and treatment strategies to be developed. Over recent years, several measurements have been described for screening frail older adults, or those in the process of becoming frail; however, none has yet been established as a gold standard. The phenotype developed by Fried, in the United States, has been highlighted as one of the most commonly used instruments in this regard.^2^^–^^4^

In searching for low-cost instruments with good applicability in clinical practice, some studies have investigated the validity of the Short Physical Performance Battery (SPPB) for screening for frailty among older adults, given the relationship between frailty and disability that has already been established.^1^^,^^5^ The SPPB is an objective, standardized, multidimensional instrument that is capable of assessing the physical performance of older adults,^6^ in addition to being useful in screening for future disabilities,^7^ frailty^8^^–^^10^ and other outcomes such as hospitalization and death.^11^ This instrument was translated and adapted for the Brazilian population by Nakano,^12^ and was found to present good reliability. However, there is a need for validation of the test using different samples of the population. The results of a study carried out among older people from different socioeconomic contexts (Brazil and Canada) revealed differences in the validity measurements for use of the SPPB between the samples, and suggested that this type of analysis is influenced by the characteristics inherent to the study population.^8^ This influence is clear in the literature, as shown through the use of different cutoff points for screening frailty among older adults from different contexts.^8^^–^^10^

When considering frailty in the Amazonian context, it is necessary to take into account the peculiarities of the region, which presents a distinct demographic transition process,^13^ large areas of demographic voids, unfavorable socioeconomic conditions and difficulty in accessing large cities, where the majority of the healthcare network is concentrated.^14^ Thus, use of easy-to-apply and low-cost measurements to screen for frailty in this context is especially relevant, and could favor implementation of strategies for prevention and treatment of this condition, thereby minimizing the occurrences of associated negative outcomes. Through using an appropriate cutoff point, it is possible that these measurements could be useful in the initial screening of older adults, for later confirmation of the diagnosis of frailty.

## OBJECTIVE

The objective of this study was to verify the validity of the SPPB as a screening tool for FS among older adults in a municipality in the interior of the state of Amazonas, Brazil.

## METHODS

This was a cross-sectional, descriptive-analytical, population-based study that used data from the “Study of Health and Frailty of the Older Adults in the Brazilian Amazon” (Estudo da Saúde e Fragilidade do Idoso da Amazônia Brasileira, ESFRIA), carried out in the municipality of Coari (AM). This project was approved by the Research Ethics Committee of the Federal University of Amazonas (CEP-UFAM) under the number 15327413.0.0000.5020, on April 18, 2013.

The study included a representative sample of older adults aged 60 years or over who were living in the urban area of the municipality of Coari. These individuals agreed to participate in the research through signing a free and informed consent statement. Individuals with any of the following conditions: cognitive impairment, identified through the Mini-Mental State Examination (MMSE), based on scores of under 13 points for illiterate older adults, 18 points for individuals with 1–7 years of education and 26 points for those with 8 or more years of education;^15^ a clinical condition that limited transference and movement; and limitations relating to physical effort. After these exclusions, the resultant sample comprised 274 older people. The characteristics of the municipality and sampling process, along with other additional information about the methodology used, were described in a previous study, by Freire Junior et al.^16^

Data collection took place between October 2013 and February 2015, in two stages. Initially, the older adults attended a structured interview at which they were asked questions relating to socioeconomic, demographic and health matters. In the second stage, they were taken to the laboratory of the Institute of Health and Biotechnology (ISB-Coari) at UFAM, where they underwent specific tests and were classified with regard to frailty using Fried's phenotype criteria, as follows:^1^

1. Unintentional weight loss: self-reported weight loss ≥ 4.5 kg or ≥ 5% of body weight in the previous year.
2. Exhaustion: self-reported via two questions from the Center for Epidemiologic Studies depression scale (CES-D): “How often in the last week did you feel that everything you did required a lot of effort?” and “How often in the last week did you feel that you could not do anything due to tiredness?”. This criterion for frailty was considered to be present in participants who answered “always” or “most of the time”.
3. Low level of physical activity: evaluated using version 8 (long) of the international physical activity questionnaire (IPAQ). The results were adjusted according to sex and the 20^th^ percentile was established as the cutoff point, namely 171.3 kcal/week for men and 87 kcal/week for women.
4. Decreased handgrip strength: evaluated by means of dynamometry (Saehan hydraulic hand dynamometer, SH5001; Masan, South Korea). This criterion was considered to be present in individuals who scored below the established cutoff points (adjusted for sex and body mass index, BMI), based on the 20^th^ percentile (worst performances for the sample).
5. Decreased gait speed: evaluated through the SPPB gait speed test. The criterion was considered to be present in individuals who performed the test in a length of time greater than the stipulated cutoff points (adjusted for sex and height).

The older adults who presented three or more of the criteria described above were considered frail; those who presented one or two criteria, pre-frail; and those who did not present any of these criteria, non-frail.^1^

To evaluate physical performance, the Brazilian version of the SPPB was used, composed of three subtests, as follows:

1. Balance test: This evaluated static balance in three standing positions: feet together; one foot partially in front of the other (*semi-tandem*); and one foot totally in front of the other (*tandem*). The older adults were required to remain in each position, looking ahead, for 10 seconds. Those who maintained balance for the necessary time in the first two positions received one point for each position. Those who were able to remain in the third position for 10 seconds received two points; those who maintained this position for 3 to 9.99 seconds received one point; and those maintained this for less than 3 seconds or who refused to try were awarded no points. The total score for the balance test was calculated by summing the points gained in each of the three positions.
2. Gait speed test: This required the participants to walk, with their usual gait, for a distance of three meters. Two attempts were timed and the shortest time obtained was used to assign the score, in accordance with the cutoff points proposed in the Brazilian version of the test.^12^
3. Chair stand test: This evaluated participants' lower-limb strength. They were asked to get up from and sit down again on a chair with a backrest, five times in a row, as quickly as possible, with the upper limbs crossed over the chest. Those who were unable to perform the test safely or who refused to take the test, along with those who failed to complete the test or completed it in more than 60 seconds, did not receive a score. The other participants received scores in accordance with the cutoff points recommended by Nakano.^12^

The total SPPB score was obtained through summing the scores obtained from each component. The total score possible ranged from 0 to 12 and was categorized as follows: 0-3 points = disability/very poor performance; 4-6 points = poor performance; 7-9 points = moderate performance; and 10-12 points = good performance.

Categorical variables were described using absolute and relative frequencies, and numerical variables using the mean and standard deviation (for age) or the median and interquartile range (for BMI and total SPPB score), according to whether the variable had normal distribution. The relationship between the total SPPB score and the frailty categories was analyzed using the Kruskal-Wallis test, and SPPB cutoff points were compared with frailty categories using the chi-square test.

The following validity measurement were used: sensitivity (proportion of individuals who truly do have frailty and present a positive test result); specificity (proportion of individuals who truly do not have frailty and present a correct negative test result); positive and negative predictive values (proportions of positive and negative results from the SPPB test that are true positive and true negative results, respectively); accuracy (proportion of individuals correctly classified as presenting frailty, among all the results); positive and negative likelihood ratios (probabilities of a positive result and of a negative result among individuals presenting frailty divided by the probabilities of a positive result and of a negative result among individuals who do not present frailty, respectively); and area under the receiver operating characteristic (ROC) curve (graphical representation of true positives plotted against false negatives).

The validity measurements were calculated for the main cutoff points of the SPPB, and served as a basis for calculating the prevalence of frailty and the post-test probability. The data were described and analyzed using the Statistical Package for the Social Sciences (SPSS) software, version 22.0 (Chicago, USA). The level of significance used in the analyses was 5% (α = 0.05) with a 95% confidence interval.

## RESULTS

Among the 274 individuals initially evaluated, a total of 10 losses were recorded due to absence of data referring to the SPPB (n = 1) or frailty (n = 9). Therefore, the current study analyzed a sample of 264 older people, with a mean age of 71.7 years (standard deviation, SD: 8), consisting mainly of women (62.5%) above ideal weight (52.6%), and who had lived for 20 years or more in riverside communities (52.7%). The illiteracy rate among the participants was 47.3%; 62.5% performed some type of subsistence activity (such as agriculture, fishing or latex extraction); and 83.7% had a family income of one or more monthly minimum wages (MW). In relation to health, more than half of these older adults (54.2%) classified their general health status as fair and 40.2% said they had three or more comorbidities (**Table 1**).

**Table 1:** Sociodemographic and health characteristics of the study sample (n = 264)

Variables		n (%)
**Age range (years)**		71.0 (12.0)*
	60-69	114 (43.2)
	70-79	106 (40.2)
	80 or over	44 (16.7)
**Sex**		
	Male	99 (37.5)
	Female	165 (62.5)
**Schooling**		
	Illiterate	125 (47.3)
	Literate or more	139 (52.7)
**Family income**		
	< 1 minimum wage	42 (15.9)
	≥ 1 minimum wage	221 (83.7)
**Time in riverside community**		
	0-19 years	122 (46.2)
	20 or more years	139 (52.7)
**Body mass index**		27.3 (6.9)*
	Malnourished	43 (16.3)
	Normal weight	82 (31.1)
	Overweight/obesity	139 (52.7)
**Number of comorbidities**		
	0-2 diseases	158 (59.8)
	3 or more diseases	106 (40.2)
**Number of drugs**		
	None	102 (38.6)
	1-2 drugs	101 (38.3)
	3 or more drugs	61 (23.1)
**Self-perceived health**		
	Very good/good	77 (29.2)
	Fair	143 (54.2)
	Poor/very poor	44 (16.7)
**Frailty classification**		
	Non-frail	82 (31.0)
	Pre-frail	157 (59.5)
	Frail	25 (9.5)

* Median (interquartile range).

The prevalence of frailty was 9.5% and the prevalence of pre-frail individuals was 59.5%, which was the highest percentage of these older adults. The median total SPPB score was 10 (IQR: 2). According to the SPPB instrument, this showed that a significant proportion of the individuals had good (63.3%) to moderate (28.4%) ability. Additional information on the distribution of the sample regarding frailty can be found in a previous published paper.^17^

**Table 2** presents the results regarding the distribution of the total SPPB score for classification of frailty and each of its components. Lower median SPPB scores were observed in the pre-frail group (10.0) and frail group (8.0), in relation to the non-frail group (11.0). Among the Fried criteria, slow gait and muscle weakness showed the worst results in relation to the SPPB scores.

**Table 2:** Characterization of the total SPPB score for the classification of frailty and for each of its components.

Total SPPB score			
**Frailty classification** *		Median (IQR)	Min-Max
	Non-frail	11.0 (2)	7-12
	Pre-frail	10.0 (3)	3-12
	Frail	8.0 (4)	4-12
**Frailty variables**		Median (IQR)	Min-Max
	Fatigue	10.0 (2)	4-12
	Weight loss	10.0 (3)	4-12
	Slow gait	7.0 (2)	3-10
	Muscle weakness	9.0 (3)	3-12
	Low level of physical activity	10.0 (2)	5-12

* P < 0.001, Kruskal-Wallis test.

SPPB = Short Physical Performance Battery; IQR = Interquartile range; Min-Max = minimum-maximum.

**Figure 1:**
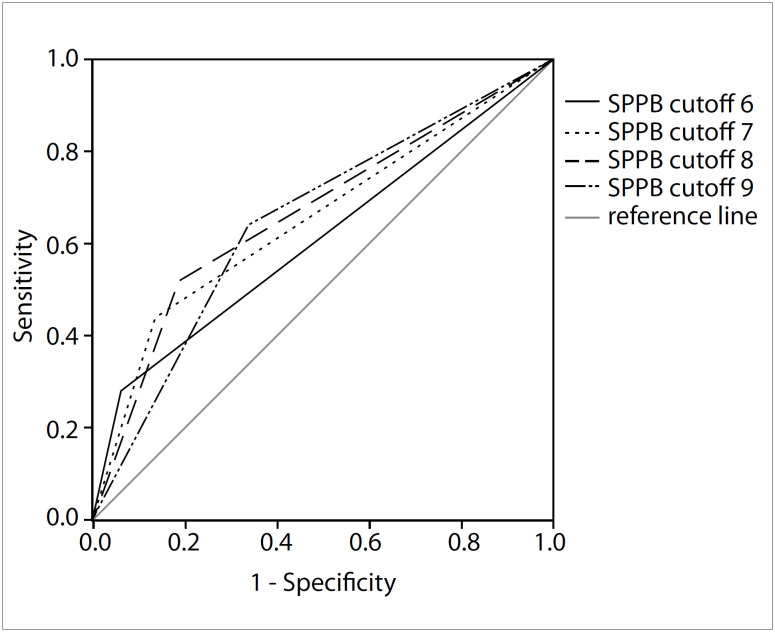
Receiver operating characteristic (ROC) curves for the Short Physical Performance Battery (SPPB) cutoff points of 6 to 9, for screening for frailty in the sample of older adults in the “Study of Health and Frailty of the Older Adults in the Brazilian Amazon” (Estudo da Saúde e Fragilidade do Idoso da Amazônia Brasileira, ESFRIA).

The sensitivity, specificity and predictive values, along with other validity measurements for each cutoff point of the total SPPB score for identifying frail older adults, are described in Table 3, in comparison with the values for non-frail and pre-frail individuals. The results from this study showed that the sensitivity values were fairly low, especially for the cutoff points of 6 (0.28) and 7 (0.44). In contrast, the specificity showed higher values as the cutoff point decreased. The highest specificity (0.94) was obtained at the cutoff point of 6, which also presented the best accuracy value (0.88), positive predictive value (0.32) and positive likelihood ratio (LR +) (4.44), in comparison with the other scores. Figure 1 graphically presents the relationship between sensitivity and specificity, using the receiver operating characteristic (ROC) curve for each cutoff point analyzed.

**Table 3:** Validity measurements for each Short Physical Performance Battery cutoff point for identifying frail older people, compared with the pre-frail and non-frail groups of the sample

Measurements	SPPB cutoff points			
	≤ 6 points (CI)	≤ 7 points (CI)	≤ 8 points (CI)	≤ 9 points (CI)
Sensitivity	0.28 (0.14-0.48)	0.44 (0.27-0.62)	0.52 (0.34-0.70)	0.64 (0.44-0.80)
Specificity	0.94 (0.90-0.96)	0.86 (0.81-0.90)	0.81 (0.75-0.85)	0.66 (0.60-0.71)
PPV	0.32 (0.16-0.53)	0.25 (0.15-0.39)	0.22 (0.14-0.35)	0.17 (0.10-0.25)
NPV	0.93 (0.89-0.95)	0.94 (0.90-0.96)	0.94 (0.90-0.35)	0.95 (0.90-0.97)
Accuracy	0.88 (0.83-0.91)	0.82 (0.77-0.86)	0.78 (0.73-0.83)	0.66 (0.60-0.71)
AUC-ROC	0.61 (0.48-0.74)	0.65 (0.53-0.78)	0.67 (0.54-0.79)	0.65 (0.54-0.77)
LR+	4.44 (2.00-9.87)	3.19 (1.85-5.50)	2.77 (1.75-4.38)	1.89 (1.34-2.66)
PTP+	0.32 (0.17-0.51)	0.25 (0.16-0.37)	0.23 (0.16-0.31)	0.17 (0.12-0.22)
LR-	0.77 (0.60-0.98)	0.65 (0.46-0.92)	0.59 (0.39-0.89)	0.54 (0.32-0.92)
PTP-	0.07 (0.06-0.09)	0.06 (0.05-0.09)	0.06 (0.04-0.09)	0.05 (0.03-0.09)
Prevalence (%)	8.3 (5.6-12.3)	16.7 (12.7-21.6)	22.0 (17.4-27.4)	36.7 (31.2-42.7)
P-value	0.002*	0.001*	0.000	0.003

* Fisher's exact test.

CI = confidence interval (95%); PPV = positive predictive value; NPV = negative predictive value; AUC-ROC = area under the receiver operating characteristic curve; LR+ = positive likelihood ratio; LR- = negative likelihood ratio; PTP+ = positive post-test probability; PTP- = negative post-test probability.

**Figure 2:**
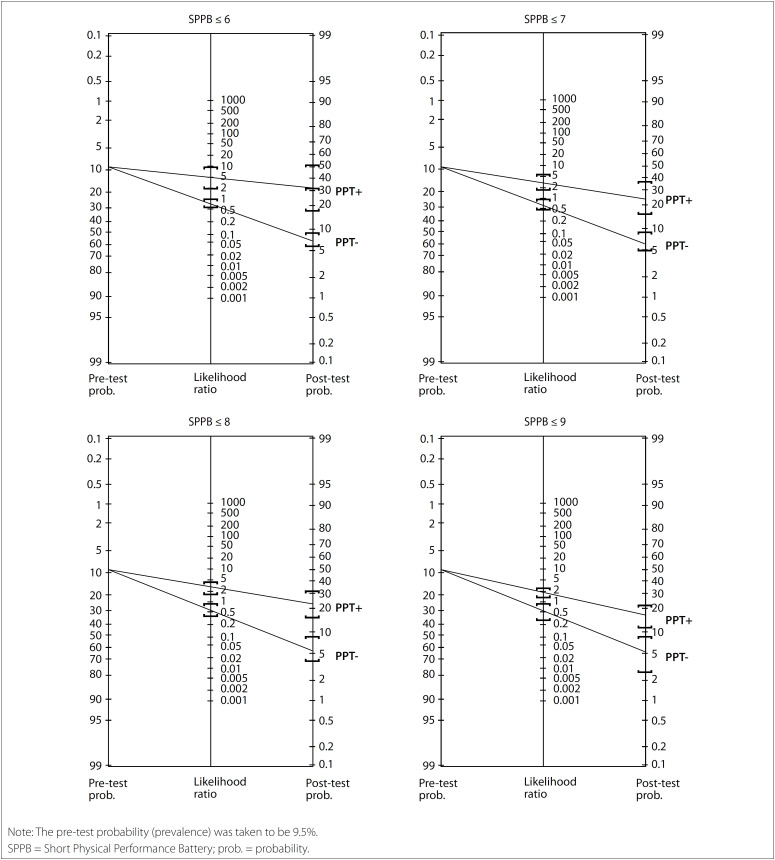
Fagan nomogram: graphical representation of the positive post-test probability (PTP+) for cutoff points of 6 to 9, for the sample of the “Study of Health and Frailty of the Older Adults in the Brazilian Amazon” (Estudo da Saúde e Fragilidade do Idoso da Amazônia Brasileira, ESFRIA).

In Figure 2, the post-test probabilities are represented by a Fagan nomogram, based on the reference prevalence (pre-test probability) and the LR + and LR- values. From the cutoff points included in the analysis, the following prevalences were calculated: 8.3% (≤ 6 points), 16.7% (≤ 7 points), 22.0% (≤ 8 points) and 36.7% (≤ 9 points).

## DISCUSSION

The current study analyzed the possible use of the SPPB as a screening tool for frailty among older adults in the municipality of Coari (AM). A strong association between low physical performance and frailty was identified in the sample studied. Among the cutoff points analyzed, the one with the best validity measurements for frailty was ≤ 6, especially regarding specificity values and positive likelihood ratios.

The condition of frailty may be present even in the absence of functional limitations.^18^ However, some studies have already demonstrated that an association exists between frailty and worse physical performance.^19^^,^^20^ The decreasing relationship between the total SPPB score and the frailty classification observed in the Coari sample is in accordance with previous studies, in which it was observed that older people with worse burden of frailty (frail and pre-frail) had worse performances in the total SPPB score than did non-frail older adults.^8^^,^^10^^,^^21^ According to Mello,^10^ from an analysis on the SPPB in relation to the frailty phenotype, the worst scores observed were in relation to the criteria of slow gait and muscle weakness. Those results were similar to what was observed in the current study. Andrade^22^ emphasized this finding through stating that frail older people can develop muscle weakness and gait changes at proportions of 3.7 and 1.7 times greater, respectively, than the risk of developing weight loss, for example.

In our analysis, the cutoff point ≤ 6 stood out as the best score for screening for frailty since, despite having low sensitivity (0.28), it demonstrated high specificity (0.94). Similar results were shown in the study by Verghese and Xue,^21^ among older Americans (70 years of age or over) living in the community, with no alteration in gait speed. Overall, they observed that lower SPPB scores demonstrated better specificity, but less sensitivity for identifying frailty. They highlighted the cutoff point ≤ 8 for the SPPB, as the most suitable for screening for frail older people in their sample, with sensitivity of 0.52 and specificity of 0.70.

Another study, carried out in Spain, in which the relationship between frailty and some functional assessment instruments was analyzed, showed that the SPPB was one of the best-performing tests for identifying frail older people. It was found that the best cutoff point was ≤ 6, with an area under the curve (AUC) of 0.956 and sensitivity and specificity of 0.88.^9^

Another study of this nature with findings similar to ours was carried out by the FIBRA Network. The results from that study showed that there was low sensitivity (0.27) and high specificity (0.85) for the cutoff point of 7. However, ≤ 8 was highlighted as the most-indicated cutoff point for positive identification of frail older adults, given that this score presented higher sensitivity (sensitivity = 0.79; specificity = 0.73).^10^

Câmara et al.^8^ suggested that the test cutoff point should be 9, since this showed moderate ability to identify frail older people in two different socioeconomic contexts: Saint Bruno, Canada, and Santa Cruz, Brazil, with better results for the Canadian sample (AUC = 0.81; sensitivity = 0.92 and specificity = 0.80).

It is known that high values for specificity in relation to sensitivity are common and desirable in screening tests or diagnostic screening, because this is useful in excluding false positives.^23^ In addition, it is common practice within clinical care to use serial tests, such that additional tests can be performed to confirm previously obtained results. Thus, the high specificity value found for the cutoff point of 6 in the Coari sample shows that the SPPB has good ability to identify individuals who are in fact not frail. Thus, this shows that it can be used as an initial screening test for the condition of frailty in that context.

In the current study, the cutoff point of 6 also presented the best accuracy value (0.88), compared with the other scores. The same cutoff point for screening for frailty was highlighted by Abizanda et al.,^9^ although with a higher accuracy value (0.96). Despite the relevance of this measurement, other statistics are needed to complement a test approach, such as predictive validity and relative risk.^24^ In our analysis on the cutoff point of 6, a high NPV (0.93) was observed, which is expected for conditions with low prevalence.^25^ This measurement indicates that the probability that the individual is not frail is 93%, after obtaining a score higher than 6 for the total SPPB score, thus indicating a highly reliable negative result. This characteristic is also common and is expected in screening tests, in order to minimize occurrences of erroneous results.^23^^,^^25^

The likelihood ratios make it possible to transform the prevalence of a condition (pre-test probability) into post-test probability.^23^ The LR+ value (4.44) and LR- value (0.77) for the cutoff point of 6 in the SPPB were the best values observed in this analysis. Mello (2015)^10^ found similar results for the SPPB cutoff point of 7 (LR+: 4.2; and LR-: 0.4). These values show that there was a small but still important change in LR+ and minimal alteration in LR-, in the post-test probability.

The calculation of the prevalence of frailty based on the cutoff point of 6 for our sample showed that the value found (8.3%) was very close to the value of the reference prevalence, obtained by means of Fried's phenotype (8.5%). The prevalence of frailty varies considerably between populations, partly due to the particularities of the study sample and partly due to the procedures used to classify older adults regarding the syndrome. Previous studies recorded higher prevalences than those found in the current study: 19.6% in Latin American countries^26^ and 13.5% in the ELSI-Brazil study.^18^ A meta-analysis that brought together studies carried out in low and middle income countries found that the prevalence of frailty ranged from 3.9% (China) to 51.4% (Cuba). In the studies that used Fried's five criteria (including measurements for weakness and slow gait), the mean rates were 12.7% for frailty and 55.2% for pre-frailty.^27^

Although an association between unfavorable socioeconomic conditions and frailty has already been demonstrated,^8^^,^^27^ there is still little research of this nature in low-income populations such as that of Coari. A national study carried out among older people in seven Brazilian municipalities with different human development indexes (HDIs) found prevalences of frailty that ranged from 7.7% to 10.8%. A rate similar to ours (9.7%) was found in the municipality of Parnaíba (PI), which was the municipality with the lowest HDI among those investigated (0.674).^28^

Despite the characteristics inherent to the Amazonian population, the findings from our study point towards some similarities between the older adults in Coari and those in other regions of Brazil and around the world, with regard to the variables analyzed here, which indicates a certain consistency of the findings. Another strength of our study is that the sampling process used enabled representative and random selection of older adults in the municipality, thus minimizing selection biases that might have influence the results. One limitation of the study was the impossibility of carrying out stratified analyses according to age group, due to the small number of individuals aged 75 years or over in the sample. Therefore, one factor that should be considered in making comparisons with other populations is that the sample of our study was composed mainly of young older people who did not have any major functional limitations and were living in the community.

## CONCLUSION

The current study demonstrated the importance and validity of the SPPB for screening for frailty syndrome among older people in an Amazonian context, especially considering its easy use within clinical care for older adults. It also confirmed that a strong association exists between frailty and low functional performance, as measured using the SPPB. A score of 6 was indicated as the best cutoff point for the population studied, with emphasis on better values of specificity, accuracy, PPV and LR+ than seen using other cutoff scores. Therefore, it is suggested that this instrument can be used in healthcare services to diagnose frailty among older people living in the Amazonian context.
